# Absence of progesterone receptor associated with secondary breast cancer in postmenopausal women

**DOI:** 10.1038/sj.bjc.6690249

**Published:** 1999-03

**Authors:** R L Balleine, M J Earl, M L Greenberg, C L Clarke

**Affiliations:** Westmead Institute for Cancer Research, Westmead Hospital, University of Sydney, Sydney, NSW, 2145, Australia; Department of Tissue Pathology, Institute of Clinical Pathology and Medical Research, Westmead, NSW, 2145, Australia

**Keywords:** breast cancer, metastatic, receptors -oestrogen -progesterone

## Abstract

The relationship between expression of receptors for oestrogen and progesterone (ER and PR) and disease progression in breast cancer was investigated by comparing immunocytochemical determinations of ER and PR in fine needle aspirates from primary and secondary breast tumours. Rates of receptor expression were significantly higher in primary than in secondary lesions: for ER 63.3% (*n* = 689) compared with 45.3% (*n* = 223), and for PR 53.7% (*n* = 443) compared with 33.1% (*n* = 121). The effect of menopausal status was examined by subdividing the patient cohort into those over or under the age of 50 years. In both instances, ER expression in secondary tumours was relatively low; however, only postmenopausal patients had significantly lower rates of PR expression in secondary tumours. Consistent with this, an increase in the ER+PR– profile in secondary tumours compared with primary cases from postmenopausal patients was seen, and in a multivariate analysis, a specific absence of PR expression in secondary tumours was revealed. Comparison of ER and PR expression in simultaneously sampled primary tumours and lymph node metastases from the same patient showed that receptor expression was stable with progression to a metastatic site as results were concordant for ER in 92% (*n* = 88) and PR in 93.8% of cases (*n* = 65). These results suggest that absence of PR expression in primary breast cancer is associated with disease progression and may be a marker of an aggressive tumour phenotype. © 1999 Cancer Research Campaign


*British Journal of Cancer* (1999) **79**(9/10), 1564–1571
© 1999 Cancer Research Campaign
Article no. bjoc.1998.0249

### Absence of progesterone receptor associated with secondary breast cancer in postmenopausal women

**RL Balleine[1], MJ Earl[2], ML Greenberg[2]* and CL Clarke[1]**

[1]Westmead Institute for Cancer Research, University of Sydney, Westmead Hospital, NSW 2145, Australia; [2]Department of Tissue Pathology, Institute of Clinical Pathology and Medical Research, Westmead Hospital, Westmead, NSW 2145, Australia

**Summary** The relationship between expression of receptors for oestrogen and progesterone (ER and PR) and disease progression in breast cancer was investigated by comparing immunocytochemical determinations of ER and PR in fine needle aspirates from primary and secondary breast tumours. Rates of receptor expression were significantly higher in primary than in secondary lesions: for ER 63.3% ($n$ = 689) compared with 45.3% ($n$ = 223), and for PR 53.7% ($n$ = 443) compared with 33.1% ($n$ = 121). The effect of menopausal status was examined by subdividing the patient cohort into those over or under the age of 50 years. In both instances, ER expression in secondary tumours was relatively low; however, only postmenopausal patients had significantly lower rates of PR expression in secondary tumours. Consistent with this, an increase in the ER+PR– profile in secondary tumours compared with primary cases from postmenopausal patients was seen, and in a multivariate analysis, a specific absence of PR expression in secondary tumours was revealed. Comparison of ER and PR expression in simultaneously sampled primary tumours and lymph node metastases from the same patient showed that receptor expression was stable with progression to a metastatic site as results were concordant for ER in 92% ($n$ = 88) and PR in 93.8% of cases ($n$ = 65). These results suggest that absence of PR expression in primary breast cancer is associated with disease progression and may be a marker of an aggressive tumour phenotype.

**Keywords:** breast cancer; metastatic; receptors -oestrogen -progesterone

The normal human breast is responsive to the ovarian steroid hormones oestrogen and progesterone and it is an essential feature of breast cancer that this hormone responsive character is frequently retained. The effects of oestrogen and progesterone are mediated by specific nuclear receptors, ER and PR. Expression of these receptors in breast tumours is associated with a number of favourable pathological features such as smaller tumour size, low tumour cell proliferation rates and low grade (Thorpe, 1988; Wenger et al, 1993), and consistent with this, an association between improved clinical outcome and receptor positivity has been reported (Pichon et al, 1996). This evidence suggests that the pattern of expression of ER and PR in breast cancer is associated with tumour subtypes which are clinically and also pathologically distinct.

The expression of ER and PR in breast cancer is a useful clinical marker of likely response to therapeutic endocrine agents (Horwitz, 1981; McGuire et al, 1991) and these receptors are therefore routinely assayed in clinical breast cancer specimens. In addition, the combined ER/PR profile of a tumour gives insight into aspects of receptor function and stimulation as the expression of PR is induced by oestrogenic stimulation of ER and, therefore, not only signals potential progesterone sensitivity, but also becomes a marker of a functioning oestrogen response pathway (Horwitz et al, 1975; Horwitz and McGuire, 1978; Clarke, 1993).

Although the predictive value of receptor status in response to endocrine agents is well established, the relationship between expression of ER and PR and tumour progression is less clear. It is not known whether tumours arise from cells with a specific receptor phenotype, for instance ER+PR+, and progressively lose first one then the other of these receptors, and receptors for other endocrine agents to eventually become receptor negative, or whether the receptor phenotype is stable as the disease progresses, with the worsening clinical course being consequent to other genetic and biological alterations.

A large number of studies have examined receptor expression in primary and secondary breast cancers, the majority of which have used biochemical assays of ER and, less frequently, PR. In general, studies on metastatic breast cancer have been limited by the low frequency with which such lesions are biopsied, so the numbers in studies published to date are limited. The trend is for the receptor phenotype of the primary and secondary tumours to be the same, although the data are complicated by the range of discordant results (Webster et al, 1978; Hoehn et al, 1979; Allegra et al, 1980; Parideans et al, 1980; Peetz et al, 1982; Harland et al, 1983; Hull et al, 1983; Gross et al, 1984; Jakesz et al, 1985; Raemakers et al, 1984; Alanko, 1985; Hahnel and Twaddle, 1985; Crawford et al, 1987, Butler et al, 1989; Spataro et al, 1992). This is probably due both to the small size of the majority of studies, as mentioned above, and to the limitations of the biochemical assays, which include admixture of non-malignant and malignant cells and the possibility that receptors bound by endogenous or pharmacological ligands are likely to be missed in ligand-binding assays of tumour receptor content (Hull et al, 1983; Encarnacion et al, 1993). More recent reports have overcome these limitations by using immunohistochemical determination of ER and PR, but the

*Received 14 April 1998*
*Revised 2 September 1998*
*Accepted 18 September 1998*

*Correspondence to:* R Balleine

*Present address: Hampson Sugarman Pathology, 151 Hawkesbury Rd, Westmead, NSW 2145, Australia numbers examined are small. Nevertheless, it appears that in matched cases the receptor phenotype of the primary cancer is likely to be maintained in the recurrent tumour in the majority of cases (Kamby et al, 1989; Muller-Holzner et al, 1993; Kuukasjarvi et al, 1996). The more general question of whether certain receptor phenotypes are associated with greater likelihood of progression to metastasis is, therefore, important.

The purpose of this study was to determine, using immunocytochemical methods in a large cohort of primary and secondary breast cancers sampled by fine needle aspiration, the combined ER and PR phenotypes, to ask whether secondary lesions are more likely to be receptor-positive than negative and to determine whether a particular ER/PR phenotype predominated in secondary lesions. The profile of receptor expression in primary and secondary tumours in pre- and postmenopausal women is not known and has also been examined in this study.

### METHODS

### Patients

Patients were women who presented for diagnostic fine needle aspiration biopsies of breast tumours to the Department of Tissue Pathology at Westmead Hospital between 1986 and 1993. ‘Primary’ tumours were aspirates of lesions in the breast diagnosed as ‘adenocarcinoma’ or ‘colloid carcinoma’. ‘Secondary’, tumours were included if the clinical details provided with the request form included a history of primary breast cancer, if there had been a previous breast aspirate or if the cytological report included a comment that the appearances were consistent with origin in breast. One additional postmenopausal patient, not meeting these criteria but with cytological and clinical features consistent with breast cancer, was also included. The ‘secondary’ tumours were diagnosed as ‘metastatic adenocarcinoma’, ‘colloid carcinoma’ or ‘adenocarcinoma’ and comprised chest wall recurrences as well as deposits in regional lymph nodes, skin and soft tissue and some visceral sites reflecting the accessibility of these to fine needle aspiration. Regional lymph node deposits contributed the largest proportion.

The cohort consisted of 807 patients from whom 927 separate fine needle aspiration biopsies of breast cancer were taken and tested for the presence of one or both receptors. The age of the patient was known at the time of 916 of these assays and ranged from 25 to 100 years. The mean age was 58.7 years. The mean age of patients with primary tumours was 59 years and secondary tumours was 57 years. In order to gauge the effect of menopausal status, women who were 50 years of age or younger at the time of the test were designated ‘premenopausal’ and those over 50 years of age, ‘postmenopausal’. The mean age of premenopausal patients with primary tumours was 43 years and 42 years for those with secondary lesions. The mean age of postmenopausal women with primary tumours was 67 years and 66 years for secondary cases.

In total there were 912 ER assays, 689 from primary tumours and 223 from secondaries, available for analysis. There were 564 PR results: 443 of these were from primary tumours and 121 from secondary deposits.

Assays performed on simultaneous aspirates from primary tumours and regional lymph node metastases were compared also. For this analysis, patients were taken from the previously described cohort, with additional cases sampled between 1993 and 1996 included to increase patient numbers. There were 88 simultaneous ER assays available for analysis and 65 cases with simultaneous PR results.

**Table 1** Multivariate analysis of the relationship between receptor expression, menopausal status and primary versus secondary tumour site

| | Odds ratio | 95% CI | *P*-value |
|---|---|---|---|
| ER | | | |
| Postmenopausal (> 50 years) | 2.5 | 1.4–4.4 | 0.002 |
| PR-positive | 62.5 | 34.3–113.9 | < 0.001 |
| Primary site | 1.0 | 0.6–1.8 | 0.956 |
| PR | | | |
| Premenopausal (≤ 50 years) | 2.7 | 1.5–4.9 | 0.001 |
| ER-positive | 63.3 | 34.6–116.0 | < 0.001 |
| Primary site | 2.7 | 1.4–5.0 | 0.002 |

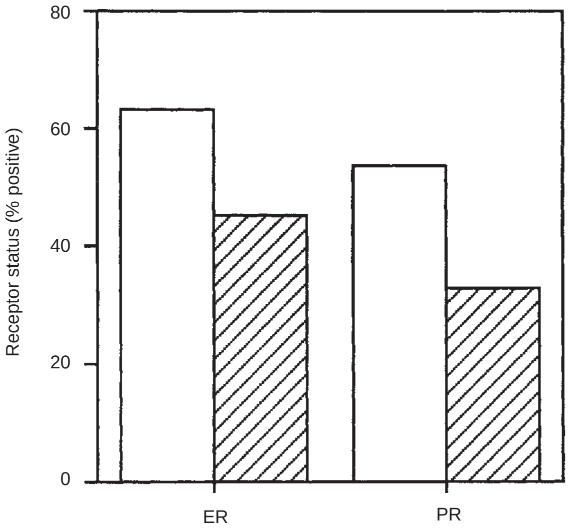

**Figure 1** ER and PR content of primary and secondary breast cancer (□): primary tumours, (▨): secondary tumours). In both instances the difference between primary and secondary cases was statistically significant, ($\chi^2$ ER: $P$ = < 0.001, PR: $P$ = < 0.001). ER primary $n$ = 689, secondary $n$ = 223. PR primary $n$ = 443, secondary $n$ = 121)

### Detection of ER and PR in fine needle aspirates by immunocytochemistry

Fine needle aspirates of breast tumours and immunocytochemical receptor determinations were performed in the Department of Tissue Pathology at Westmead Hospital as has previously been described (Greenberg et al, 1989). Briefly: aspirated material was fixed in freshly prepared 10% formalin in 0.01 M phosphate-buffered saline (PBS), at 4°C for 10–30 min after which time four cytospin slides were prepared. Chrome alum-gelatin coated slides were used to improve cell adhesion. The slides were then stored in chilled storage medium at –10°C to –20°C for up to 4 months. Prior to staining, a test and control slide were rinsed in two changes of PBS pH 7.3 for 10 min to remove storage medium. The slides were placed in 100% methanol at –15°C for 5 min followed by acetone at –15°C for 3 min. The slides were rinsed in PBS. Immunostaining was performed using the Abbott ER-ICA and PgR-ICA kits (Abbott Laboratories, Diagnostics Division, USA), according to the manufacturer’s instructions. A positive control

© Cancer Research Campaign 1999

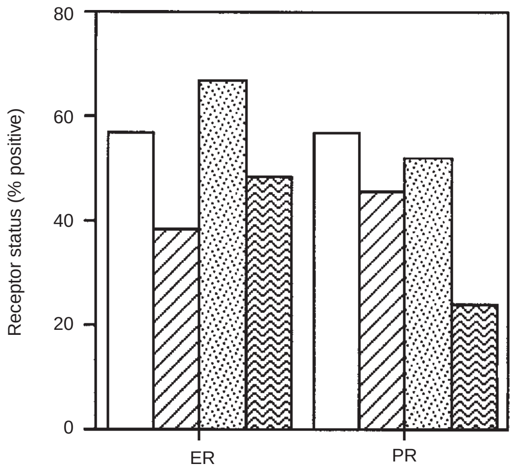

**Figure 2** ER and PR content of primary and secondary breast cancer: effect of menopausal status (□: premenopausal primary tumours, ▨: premenopausal secondary tumours, ▩: postmenopausal primary tumours, ▩: postmenopausal secondary tumours). In both age groups, primary tumours were more likely to be ER-positive than secondary tumours, ($\chi^2$ premenopausal $P$ = 0.005, postmenopausal $P$ = < 0.001). In premenopausal patients, there was no significant difference in PR status between primary and secondary tumours, whereas in postmenopausal patients secondary tumours were more likely to be PR-negative ($\chi^2$ premenopausal $P$ = 0.178, postmenopausal $P$ = < 0.001). Premenopausal primary ER $n$ = 227, PR $n$ = 151. Premenopausal secondary ER $n$ = 78, PR $n$ = 46. Postmenopausal primary ER $n$ = 453, PR $n$ = 287. Postmenopausal secondary ER $n$ = 144, PR $n$ = 74

was included in each staining run and negative controls comprised a slide from each case in which the primary antibody was substituted with normal rat antibody supplied with the kit.

Immunocytochemical smears were evaluated by light microscopy at × 400 magnification. A minimum of 200 tumour cell nuclei were counted and cases were designated 'unsatisfactory' if fewer cells were available for assessment. A scoring system from 0 to 6, which combined the percentage of nuclei stained and an estimate of the predominant nuclear staining intensity, was used to report results (Greenberg et al, 1989). On the basis of previous comparisons of immunocytochemical score and assays of receptor content in breast tumour cytosol preparations (Greenberg et al, 1989; MJ Earl, unpublished observations), scores of 0, 1 or 2 were regarded as 'negative' and scores of 3 or higher as 'positive'. For ER and PR in both primary and secondary tumours over 90% of cases designated 'negative' had immunocytochemical scores of 0.

### Statistical methods

Chi-squared tests were performed using Abacus Concepts, StatView Student software (Abacus Concepts Inc., Berkeley, CA, USA). For simultaneously sampled primary and regional lymph node metastases, the McNemars test was used to test whether the distribution of discordant cases, between instances where the primary tumour was receptor-positive and the secondary was recepter-negative and vice versa, was significantly different from random. The McNemars test was done using ARCUS Professional version 1.00S software (© Iain Buchan 1990). The multivariate analysis was done using SPSS for Windows, Release 5.0.1, statistical analysis software.

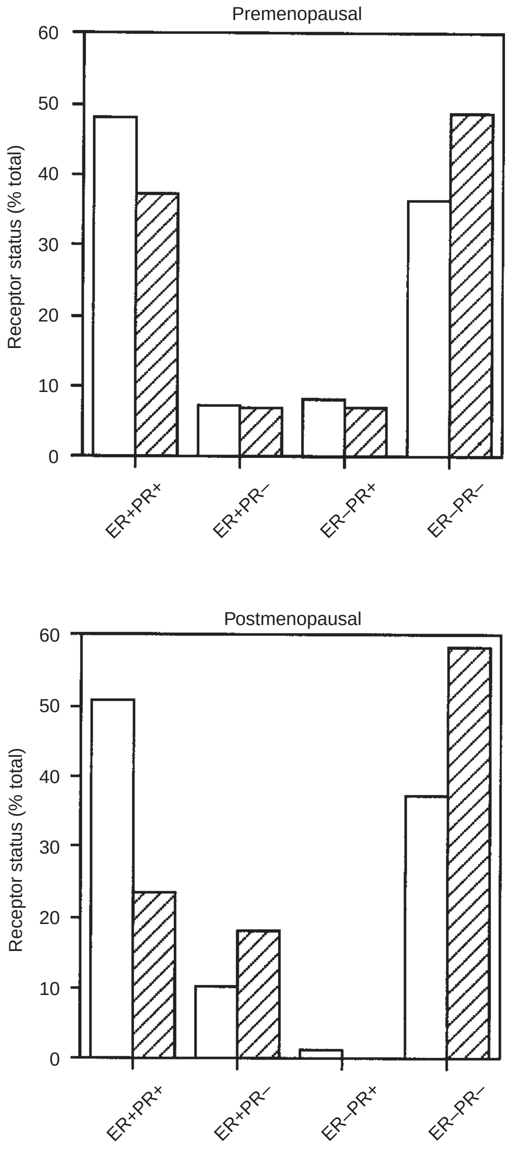

**Figure 3** Combined ER and PR phenotype according to menopausal status (□: primary tumours, ▨: secondary tumours). Premenopausal primary $n$ = 148, secondary $n$ = 43. Postmenopausal primary $n$ = 281, secondary $n$ = 72

### RESULTS

### ER and PR in primary and secondary breast cancer

Overall, 63.3% of primary tumours were ER-positive compared with 45.3% of secondary tumours and 53.7% of primaries were PR-positive compared with 33.1% of secondaries (Figure 1). In both instances, $\chi^2$ tests showed the difference between the primary and secondary tumour groups to be highly statistically significant. The data show that secondary lesions are significantly less likely to contain receptors, suggesting either that primary tumours lacking receptors are more likley to metastasize or, a less likely explanation based on published studies, that receptor-positive primaries become receptor-negative on disease progression.

*© Cancer Research Campaign 1999*

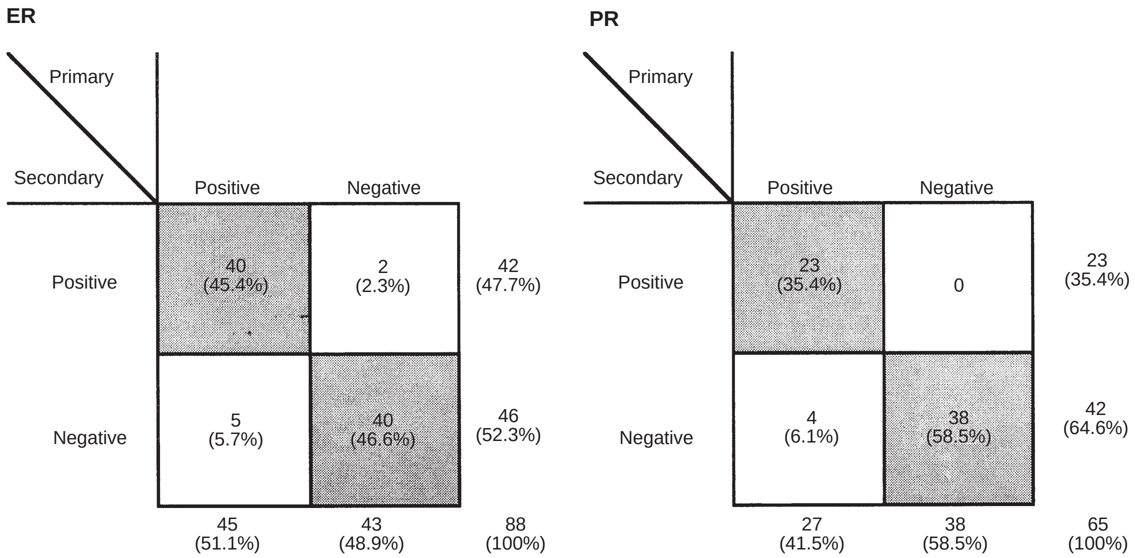

**Figure 4** Simultaneous ER and PR assays on primary breast cancer and regional lymph node metastases

### Effect of menopausal status on hormone receptor expression

The cohort was subdivided into women 50 years of age and younger and those over the age of 50 in order to gauge the effect of menopausal status (Figure 2). Amongst older women, 66.9% of primary tumours were ER-positive compared with 48.6% of secondary deposits. In the younger patient subgroup, 56.8% of primary tumours were ER-positive compared with 38.5% of secondary lesions. Overall, older women were more likely to have ER-positive tumours. In both age groups, by $\chi^2$ analysis, primary tumours were significantly more likely to be ER-positive than secondaries.

In contrast, primary tumours from younger women showed slightly higher rates of PR positivity than those from older women. Although there was a modest drop in the rate of PR expression in secondary tumours compared with primaries in the younger patient subpopulation, from 57.0% to 45.7%, this did not achieve statistical significance. In older patients, however, the rate of PR expression was markedly lower in secondary tumours, only 24.3% of cases being positive compared with 52.3% of primaries.

Examination of the combined expression of ER and PR revealed that the most common receptor phenotypes were a presence or absence of both receptors, illustrating the fact that the expression of ER and PR were closely related to each other. When the population was subdivided into pre- and postmenopausal patients, striking differences in the profiles of ER and PR expression in primary and secondary tumours were revealed (Figure 3). Amongst premenopausal women, ER/PR phenotypes were similar in primary and secondary lesions. In the postmenopausal subgroup, primary tumours were predominantly ER+PR+ and there was a marked drop in the incidence of this receptor phenotype amongst secondary tumours which was accounted for not only by an increase in the number of ER–PR– cases but also a relative increase in ER+ PR– cases. Thus secondary tumours from older women were usually PR–, even if ER expression was retained.

The observation that there was a specific lack of PR expression in the secondary tumours of postmenopausal women was tested by multivariate analysis including receptor status, menopausal status and whether a tumour was primary or secondary (Table 1). When these variables were taken into account, with respect to ER it was found that postmenopausal patients were more likely to have ER-positive tumours and ER expression was closely related to the presence of PR such that the odds of having an ER-positive tumour were almost 63 times higher if PR was present. However, when corrected for age and PR status, there was no longer a significant difference between the rates of ER positivity in primary and secondary tumours ($P$ = 0.956). In the analysis of PR, premenopausal patients were more likely to have PR-positive tumours and again the strong relationship between ER and PR expression was demonstrated. Importantly, however, PR expression was still significantly more common in primary than secondary tumours even when age and ER status were taken into account ($P$ = 0.002).

### Simultaneous receptor assays on primary tumours and corresponding regional lymph node metastases

In order to determine whether the lower rates of expression of ER and PR in secondary with respect to primary tumours (Figures 1 and 2) reflected loss or alteration of receptor phenotype upon progression to a metastatic site in this patient cohort, the receptor content of primary tumours and simultaneously sampled corresponding regional lymph node metastases were compared.

For ER, 88 cases were available for analysis (Figure 4). In 81 (92%) of these the ER status of both the primary and metastatic tumour were the same. There were five cases (5.7%) of ER+ primaries associated with ER– metastases and two cases (2.3%) where the primary was ER– but the lymph node deposit ER+. On the basis of these results there was no evidence that ER expression was systematically lost with progression from a primary to a metastatic site (McNemars test, $P$ = 0.3).

In respect of PR, there were 65 cases available for study (Figure 4). In 61 (93.8%) of these there was concordance between the PR status of the primary tumour and the regional lymph node metastasis. The four discordant cases were all PR+ primaries associated

© Cancer Research Campaign 1999

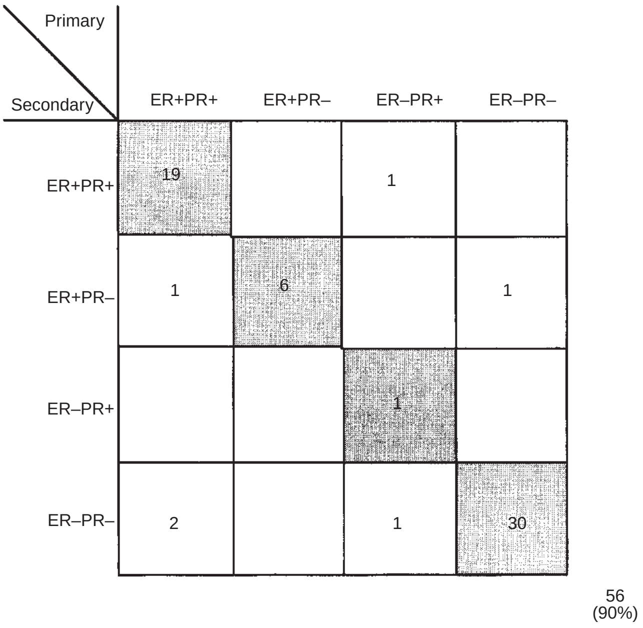

**Figure 5** Combined ER and PR phenotype of primary tumours and regional lymph node metastases with PR– metastases. Thus 4/27 (14.8%) of PR+ primary tumours were associated with PR– metastases (McNemars test, $P = 0.045$). There was some evidence therefore that, although PR status was consistent in primary and metastatic lesions, in a small number of cases PR expression may be lost with progression to a metastatic site. When this cohort was subdivided into patients under or over the age of 50 years, two of the discordant cases fell into each category.

In order to investigate whether the specific lack of PR in metastases from postmenopausal women (Figure 3, Table 1) arose in the context of an alteration in the combined ER/PR phenotype upon progression to a metastatic site, combined ER and PR status was compared in 62 corresponding primary tumours and metastases (Figure 5). The concordance between primary and secondary tumours was high, with the same receptor phenotype being found in 56 of 62 cases examined (90.3%). Notably, the ER+PR– phenotype was present in six primary tumours, and in all of these cases this receptor profile was maintained in the metastatic lesion. There were no postmenopausal patients with ER+PR+ primary tumours associated with ER+PR– metastases (data not shown). Taken together, the data support the view that the receptor phenotype was stable with progression to a metastatic site and, therefore, that the lack of PR observed in secondary tumours in postmenopausal women may be attributed to a greater tendency for PR– primary tumours to metastasize.

### DISCUSSION

There is considerable variability in the reported rates of ER and PR expression in primary breast cancer, for example in three large series, ER positivity ranged from 68.8 to 81% and PR from 54 to 70% of cases (Thorpe and Rose, 1986; Wenger et al, 1993; Pichon et al, 1996). In this cohort, with 62.4% of tumours being ER-positive and 53.2% expressing PR, the proportion of receptor-positive tumours was relatively low. A possible explanation for this is that tumours that are amenable to fine needle aspiration are likely to be relatively large and an inverse relationship between receptor expression and tumour size has been reported (Clark et al, 1984; Thorpe and Rose, 1986; Pichon et al, 1996). In other respects, however, the characteristics of receptor expression in primary tumours in this population were typical: older women were more likely to have ER-positive breast cancer than younger women and, conversely, the rate of PR positivity was higher in younger women, which is consistent with other reports (Clark et al, 1984; Thorpe and Rose, 1986; Romain et al, 1995). Similarly, there was a distinct differential distribution of the discordant receptor phenotypes with ER+PR– tumours found principally in older women and ER–PR+ tumours in younger patients (Osborne et al, 1980; Thorpe, 1988; Bonnier et al, 1995).

The profile of expression of receptors in secondary tumours was markedly different than that seen in primary cases. Secondary tumours were significantly less likely to be ER+ than primary tumours and in the subgroup of women over 50 years, PR expression was also significantly less common. Multivariate analysis of these results revealed a specific lack of PR expression in secondary tumour deposits compared with primary tumours. This result reflects the increased prevalence of the ER+PR– phenotype amongst secondary tumours in postmenopausal patients.

It emerges from this analysis, therefore, that secondary tumour deposits from postmenopausal patients are unlikely to be PR+ even if ER is present. The failure of secondary tumour deposits to express receptors, in particular PR, may indicate that primary tumours that are receptor-negative are more likely to progress and, therefore, become over-represented in the 'secondary' subgroup, or else that receptor expression is lost as a tumour progresses to a secondary site, over time or under the influence of therapy.

The issue of the stability of receptor expression with progression to metastatic sites was addressed by comparing results of ER and PR assays from simultaneous aspirates performed on primary tumours and regional lymph node metastases. For ER, the two assays were concordant in 92.0% of cases. This result is consistent with reports in the literature that have found the ER status of primary tumours to be concordant with lymph node or skin deposits in 81–94% of cases (Hoehn et al, 1979; Jakesz et al, 1985; Hahnel and Twaddle, 1985; Butler et al, 1989; Kamby et al, 1989; Kuukasjarvi et al, 1996). Amongst the small number of cases with discordant results, there was no evidence that ER expression was lost with metastasis.

Simultaneous assays of PR in primary and regional lymph node deposits from 65 patients were concordant in 93.8% of cases. All of the discordant cases were PR+ primary tumours associated with PR– metastases, indicating that PR is lost upon progression in some cases. This is consistent with published series, which have reported concordant PR results in primary tumours and lymph node or other metastases in 76–91% of cases although the number of patients in these studies tends to be small (range 18–111, mean 41), (Gross et al, 1984; Jakesz et al, 1985; Alanko, 1985; Butler et al, 1989; Kuukasjarvi et al, 1996). In most reports it was more common for the primary tumour to be PR-positive and the metastasis negative than the reverse (Jakesz et al, 1985; Butler et al, 1989; Kuukasjarvi et al, 1996). The available evidence, therefore, supports the view that PR expression is stable with disease progression to a metastatic site; however, may be lost in this process in a small number of cases.

© Cancer Research Campaign 1999

The combined ER/PR phenotype of the primary tumours and regional lymph node metastases was concordant in 90.3% of the 62 cases examined. In relation to the relatively high frequency of the ER+PR– receptor phenotype amongst secondary tumours, it is noted that there was only one case of an ER+PR+ primary associated with an ER+PR– metastasis, indicating the ER+PR– cases are unlikely to evolve from ER+PR+ tumours. There were six cases where tumour in the breast and lymph node were both ER+PR–, supporting the view that this phenotype is stable.

The possibility that receptor expression is lost over time has been examined in a number of studies which have documented sequential assays from patients who have not been given interval systemic therapy. These have returned conflicting results; however, on balance there is little evidence to support a progressive loss of ER expression over time (Webster et al, 1978; Allegra et al, 1980; Paridaens et al, 1980; Peetz et al, 1982; Hull et al, 1983; Jakesz et al, 1985; Crawford et al, 1987; Spataro et al, 1992; Kuukasjarvi et al, 1996). There is less information pertaining to PR, but in three relatively small studies, there was support for the view that PR expression may be lost over time in some tumours (Gross et al, 1984; Jakesz et al, 1985; Kuukasjarvi et al, 1996). Clearly, larger studies are needed to settle this issue but at present there is consensus in the finding that ER and PR expression are stable over time in the majority of cases.

It is possible that PR expression may be lost in metastatic lesions due to the effects of treatment, and there is evidence from a number of small studies that a transition from receptor-positive to -negative following endocrine therapy may occur (Hull et al, 1983; Gross et al, 1984; Nomura et al, 1985; Encarnacion et al, 1993). Johnston et al (1995) reported a reduction in the rate of ER positivity from 51% to 29% in 72 breast tumours treated with tamoxifen which had acquired resistance or else were resistant de novo. In 34 cases which relapsed during adjuvant tamoxifen therapy there was also a significant reduction in PR expression. Prior systemic therapy may be implicated, therefore, in the tendency for secondary tumours to be receptor-negative and may explain the lack of PR in some secondaries from postmenopausal women. Our observation from simultaneously sampled primary and secondary lesions, that ER+PR– secondary tumours were mostly associated with the same receptor profile in the primary implies, however, that the role, if any, of interval therapy in the prevalence of ER+PR– secondary tumours is likely to be minor.

The expression of PR is induced by stimulation of the ER by oestrogen (Horwitz and McGuire, 1978; Clarke, 1993), and therefore failure of a proportion of ER+ tumours in postmenopausal patients to also express PR may simply be a consequence of a low oestrogen environment providing insufficient stimulation for PR induction. In this case ER+PR– tumours would not be functionally different from ER+PR+ cases. There is evidence, however, that, although plasma oestradiol is relatively low in postmenopausal women, the hormone is concentrated in breast tissue and is present in similar levels in pre- and postmenopausal patients (van Landeghem et al, 1985). Indeed, Saez et al (1978) reported that plasma levels of oestradiol and progesterone were similar in postmenopausal patients with ER+PR+ tumours compared with those with ER+PR– tumours. Further evidence in favour of ER+PR– breast cancer being distinct is that it can be distinguished clinically from ER+PR+ disease. The rate of response to endocrine therapy is much lower, being approximately 38% in ER+PR– cases and 74% in ER+PR+ cases (Horwitz, 1981), and disease-free survival is also shorter if PR is not expressed (Clark et al, 1983). The oestrogen-dependent nature of PR expression also raises the possibility that failure of PR expression may be a consequence of aberrant ER function. Using PR as a marker of ER function was the original rationale for measuring PR routinely in breast cancer (Horwitz et al, 1975), and the increased rate of response to endocrine therapy when both receptors are present is indirect support for this hypothesis. The ER+PR– receptor profile amongst a proportion of tumours may, therefore, signal an association between ER dysfunction and disease progression.

The absence of PR expression in some breast tumours may also be due to deletion of the PR gene. The PR gene is located at chromosome 11q22 (Mattei et al, 1988), which is an area of common loss in breast cancer (Carter et al, 1994; Hampton et al, 1994; Tomlinson et al, 1996) and an association between loss of heterozygosity in this region and failure of PR expression in primary breast cancer has been reported (Tomlinson et al, 1996). Failure of PR expression may, therefore, reflect a specific pattern of genetic abnormality in breast cancer and may be a marker of molecular derangement which is associated with the likelihood of disease progression.

There are cogent reasons why tumours which fail to express PR may be biologically distinct and may be associated with an increased tendency to metastasize. In the normal breast and in cultured breast cancer cells progesterone has profound effects on cell growth and function (Graham and Clarke, 1997). One of the important physiological roles of progesterone is in limiting the action of oestrogen, which in the normal uterus occurs consequent to progesterone down-regulation of ER levels and induction of enzymes which metabolize oestrogen to products with lower oestrogenic activity (Clarke and Sutherland, 1990). Progestin down-regulation of ER and inhibition of oestrogenic activity has also been demonstrated convincingly in cultured breast cancer cells (Clarke and Sutherland, 1990). Furthermore, more recent evidence has shown in vitro that one of the isoforms of PR, PR A, can inhibit the action of ER directly (Chalbos and Galtier, 1994; McDonnell and Goldman, 1994; Kraus et al, 1995, 1997). Taken together, an important role of progesterone, and by extension PR, in breast cancer may be to inhibit the action of oestrogen and thereby limit its known tumour growth promoting effects. A tumour which lacks PR would lack this oestrogen-limiting capacity and this may be clinically associated with an increased tendency to metastasize.

In summary, a comparison of receptor expression in primary and secondary breast cancer deposits has shown that secondary tumours were likely to be receptor-negative and in older women, PR was uncommonly present even if ER expression was retained. The lack of PR in secondary lesions could not be attributed to loss of receptor expression with progression from the primary to a metastatic site which was consistent with the view that PR-negative primary tumours were more likely to progress than those which contain PR. These data suggest that failure of PR expression may be associated with an aggressive breast tumour phenotype and may have implications for disease progression. This conclusion is in accordance with the association between PR expression and both hormone responsiveness and improved survival in breast cancer. It is unclear, however, whether there is a causal relationship between disease progression and failure of PR expression, or whether PR negativity is more a marker of molecular aberrations within a tumour which determine a particular clinical course. Tumours with the ER+PR– receptor profile, which emerges as an important subcategory in this analysis, may give insight into the

© Cancer Research Campaign 1999

mechanism by which PR expression is lost and the pathological significance of this process.

### ACKNOWLEDGEMENTS

This work was undertaken with the support of the Departments of Radiation Oncology and Medical Oncology at Westmead Hospital. Advice on statistical aspects of the project was given by Dr Karen Bythe, Consultant Statistician, Clinical Sciences, Westmead Hospital. RLB and CLC gratefully acknowledge the support of the National Health and Medical Research Council of Australia, the Leo and Jenny Leukaemia and Cancer Foundation and the University of Sydney Cancer Research Fund.

### REFERENCES

Alanko A (1985) Variation of estrogen and progesterone receptor status in breast cancer. *Ann Clin Res* **17**: 10–14

Allegra JC, Barlock A, Huff KK and Lippman ME (1980) Changes in multiple or sequential estrogen receptor determinations in breast cancer. *Cancer* **45**: 792–794

Bonnier P, Romain S, Charpin C, Lejeune C, Tubiana N, Martin P-M and Piana L (1995) Age as a prognostic factor in breast cancer: relationship to pathologic and biologic factors. *Int J Cancer* **62**: 138–144

Butler JA, Trezona T, Vargas H and State D (1989) Value of measuring hormone receptor levels of regional metastatic carcinoma of the breast. *Arch Surg* **124**: 1131–1135

Carter SL, Negrini M, Baffa R, Gillum DR, Rosenberg AL, Schwartz GF and Croce CM (1994) Loss of heterozygosity at 11q22–q23 in breast cancer. *Cancer Res* **54**: 6270–6274

Chalbos D and Galtier F (1994) Differential effect of forms A and B of human progesterone receptor on estradiol-dependent transcription. *J Biol Chem* **269**: 23007–23012

Clarke CL (1993) Ovarian steroid hormone receptors and their mechanisms of action. In *Molecular Aspects of Placental and Fetal Membrane Autocoids*, Rice GE and Brennecke SP (eds), pp. 27–54. CRC Press: Boca Raton

Clarke CL and Sutherland RL (1990) Progestin regulation of cellular proliferation. *Endocr Rev* **11**: 266–301

Clark GM, McGuire WL, Hubay CA, Pearson OH and Marshall JS (1983) Progesterone receptors as a prognostic factor in stage II breast cancer. *N Engl J Med* **309**: 1343–1347

Clark GM, Osborne CK and McGuire WL (1984) Correlations between estrogen receptor, progesterone receptor, and patient characteristics in human breast cancer. *J Clin Oncol* **2**: 1102–1109

Crawford DJ, Cowan S, Fitch R, Smith DC and Leake RE (1987) Stability of oestrogen receptor status in sequential biopsies from patients with breast cancer. *Br J Cancer* **56**: 137–140

Encarnacion CA, Ciocca DR, McGuire WL, Clark GM, Fuqua SAW and Osborne CK (1993) Measurement of steroid hormone receptors in breast cancer patients on tamoxifen. *Breast Cancer Res Treat* **26**: 237–246

Graham JD and Clarke CL (1997) Physiological action of progesterone in target tissues. *Endocr Rev* **18**: 502–519

Greenberg ML, Earl MJ, Bilous AM, Ekberg H, Milliken J and Pacey NF (1989) Estrogen receptor immunocytochemical assay on cytologic material from primary and metastatic breast cancer. *Pathology* **21**: 93–99

Gross GE, Clark GM, Chamness GC and McGuire WL (1984) Multiple progesterone receptor assays in human breast cancer. *Cancer Res* **44**: 836–840

Hahnel R and Twaddle E (1985) The relationship between estrogen receptors in primary and secondary breast carcinomas and in sequential primary breast carcinomas. *Breast Cancer Res Treat* **5**: 155–163

Hampton GM, Mannermaa A, Winquist R, Alavaikko M, Blanco G, Taskinen PJ, Kiviniemi H, Newsham I, Cavenee WK and Evans GA (1994) Loss of heterozygosity in sporadic human breast carcinoma: a common region between 11q22 and 11q23.3. *Cancer Res* **54**: 4586–4589

Harland RNL, Barnes DM, Howell A, Ribeiro GG, Taylor J and Sellwood RA (1983) Variation of receptor status in cancer of the breast. *Br J Cancer* **47**: 511–515

Hoehn JL, Plotka ED and Dickson KB (1979) Comparison of estrogen receptor levels in primary and regional metastatic carcinoma of the breast. *Ann Surg* **190**: 69–71

Horwitz KB (1981) Is a functional estrogen receptor always required for progesterone receptor induction in breast cancer? *J Steroid Biochem* **15**: 209–217

Horwitz KB and McGuire WL (1978) Estrogen control of progesterone receptor in human breast cancer. Correlation with nuclear processing of the estrogen receptor. *J Biol Chem* **253**: 2223–2228

Horwitz KB, McGuire WL, Pearson OH and Segaloff A (1975) Predicting response to endocrine therapy in human breast cancer a hypothesis. *Science* **189**: 726–727

Hull DF III, Clark GM, Osborne CK, Chamness GC, Knight III WA and McGuire WL (1983) Multiple estrogen receptor assays in human breast cancer. *Cancer Res* **43**: 413–416

Jakesz R, Dittrich C, Hanusch J, Kolb R, Lenzhofer R, Moser K, Rainer H, Reiner G, Schemper M, Spona J and Teleky B (1985) Simultaneous and sequential determinations of steroid hormone receptors in human breast cancer. *Ann Surg* **201**: 305–310

Johnston SRD, Saccani-Jotti G, Smith IE, Salter J, Newby J, Coppen M, Ebbs SR and Dowsett M (1995) Changes in estrogen receptor, progesterone receptor, and pS2 expression in tamoxifen-resistant human breast cancer. *Cancer Res* **55**: 3331–3338

Kamby C, Bruun Rasmussen B and Kristensen B (1989) Oestrogen receptor status of primary breast carcinomas and their metastases. Relation to pattern of spread and survival after recurrence. *Br J Cancer* **60**: 252–257

Kraus WL, Weis KE and Katzenellenbogen BS (1995) Inhibitory cross-talk between steroid hormone receptors: differential targeting of estrogen receptor in the repression of its transcriptional activity by agonist- and antagonist-occupied progestin receptors. *Mol Cell Biol* **15**: 1847–1857

Kraus WL, Weis KE and Katzenellenbogen BS (1997) Determinants for the repression of estrogen receptor transcriptional activity by ligand-occupied progestin receptors. *J Steroid Biochem Mol Biol* **63**: 175–188

Kuukasjarvi T, Kononen J, Helin H, Holli K and Isola J (1996) Loss of estrogen receptor in recurrent breast cancer is associated with poor response to endocrine therapy. *J Clin Oncol* **14**: 2584–2589

Mattei M-G, Krust A, Stropp U, Mattei J-F and Chambon P (1988) Assignment of the human progesterone receptor to the q22 band of chromosome 11. *Hum Genet* **78**: 96–97

McDonnell DP and Goldman ME (1994) RU486 exerts antiestrogenic activities through a novel progesterone receptor A-form mediated mechanism. *J Biol Chem* **269**: 11945–11949

McGuire WL, Chamness GC and Fuqua SAW (1991) Estrogen receptor variants in clinical breast cancer. *Mol Endocrinol* **5**: 1571–1577

Muller-Holzner E, Zeimet AG, Daxenbichler G, Marth C, Muller LC and Dapunt O (1993) Progesterone receptors in routinely paraffin-embedded primary breast carcinomas and lymph node metastases. *Breast Cancer Res Treat* **25**: 47–55

Nomura Y, Tashiro H and Shinozuka K (1985) Changes of steroid hormone receptor content by chemotherapy and/or endocrine therapy in advanced breast cancer. *Cancer* **55**: 546–551

Osborne CK, Yochmowitz MG, Knight WA and McGuire WL (1980) The value of estrogen and progesterone receptors in the treatment of breast cancer. *Cancer* **46**: 2884–2888

Paridaens R, Sylvester RJ, Ferrazzi E, Legros N, Leclercq G and Heuson JC (1980) Clinical significance of the quantitative assessment of estrogen receptors in advanced breast cancer. *Cancer* **46**: 2889–2895

Peetz ME, Nunley DL, Moseley HS, Keenan EJ, Davenport CE and Fletcher WS (1982) Multiple simultaneous and sequential estrogen receptor values in patients with breast cancer. *Am J Surg* **143**: 591–594

Pichon MF, Broet P, Magdelenat H, Delarue JC, Spyratos F, Basuyau JP, Saez S, Rallet A, Courriere P, Millon R and Asselain B (1996) Prognostic value of steroid receptors after long term follow-up of 2257 operable breast cancers. *Br J Cancer* **73**: 1545–1551

Raemaekers JM, Beex LV, Koenders AJ, Pieters GF, Smals AG, Benraad TJ and Kloppenborg PW (1984) Concordance and discordance of estrogen and progesterone receptor content in sequential biopsies of patients with advanced breast cancer:relation to survival. *Eur J Cancer Clin Oncol* **20**: 1011–1018

Romain S, Laine Bidron C, Martin PM and Magdelenat H (1995) EORTC Receptor Study Group Report: steroid receptor distribution in 47 892 breast cancers. A collaborative study of 7 European laboratories. *Eur J Cancer* **31A**: 411–417

Saez S, Martin PM and Chouvet CD (1978) Estradiol and progesterone receptor levels in human breast adenocarcinoma in relation to plasma estrogen and progesterone levels. *Cancer Res* **38**: 3468–3473

Spataro V, Price K, Goldhirsch A, Cavalli F, Simoncini E, Castiglione M, Rudenstam C-M, Collins J, Lindtner J and Gelber RD (1992) Sequential estrogen receptor determinations from primary breast cancer and at relapse: prognostic and therapeutic relevance. *Ann Oncol* **3**: 733–740

 *© Cancer Research Campaign 1999*

Thorpe SM (1988) Estrogen and progesterone receptor determinations in breast cancer: technology, biology and significance. *Acta Oncol* **27**: 1–19
Thorpe SM and Rose C (1986) Oestrogen and progesterone receptor determinations in breast cancer: technology and biology. *Cancer Surv* **5**: 505–525
Tomlinson IPM, Nicolai H, Solomon E and Bodmer WF (1996) The frequency and mechanism of loss of heterozygosity on chromosome 11q in breast cancer. *J Pathol* **180**: 38–43
van Landeghem AAJ, Poortman J, Abuurs M and Thijssen JHH (1985) Endogenous concentration and subcellular distribution of estrogens in normal and malignant human breast tissue. *Cancer Res* **45**: 2900–2906
Webster DJT, Bronn DG and Minton JP (1978) Estrogen receptor levels in multiple biopsies from patients with breast cancer. *Am J Surg* **136**: 337–338
Wenger CR, Beardslee S, Owens MA, Pounds G, Oldaker T, Vendely P, Pandian MR, Harrington D, Clark GM and McGuire WL (1993) DNA ploidy, S-phase, and steroid receptors in more than 127,000 breast cancer patients. *Breast Cancer Res Treat* **28**: 9–26

© Cancer Research Campaign 1999
